# A Machine Learning Model Ensemble for Mixed Power Load Forecasting across Multiple Time Horizons

**DOI:** 10.3390/s23125436

**Published:** 2023-06-08

**Authors:** Nikolaos Giamarelos, Myron Papadimitrakis, Marios Stogiannos, Elias N. Zois, Nikolaos-Antonios I. Livanos, Alex Alexandridis

**Affiliations:** 1 Department of Electrical and Electronic Engineering, University of West Attica, Thivon 250, 122 41 Aigaleo, Greece; 2 EMTECH SPACE P.C., Korinthou 32 & S. Davaki, Metamorfosi, 144 51 Athens, Greece

**Keywords:** load forecasting, ensemble learning, neural networks, sparse representation, support vector regression

## Abstract

The increasing penetration of renewable energy sources tends to redirect the power systems community’s interest from the traditional power grid model towards the smart grid framework. During this transition, load forecasting for various time horizons constitutes an essential electric utility task in network planning, operation, and management. This paper presents a novel mixed power-load forecasting scheme for multiple prediction horizons ranging from 15 min to 24 h ahead. The proposed approach makes use of a pool of models trained by several machine-learning methods with different characteristics, namely neural networks, linear regression, support vector regression, random forests, and sparse regression. The final prediction values are calculated using an online decision mechanism based on weighting the individual models according to their past performance. The proposed scheme is evaluated on real electrical load data sensed from a high voltage/medium voltage substation and is shown to be highly effective, as it results in R^2^ coefficient values ranging from 0.99 to 0.79 for prediction horizons ranging from 15 min to 24 h ahead, respectively. The method is compared to several state-of-the-art machine-learning approaches, as well as a different ensemble method, producing highly competitive results in terms of prediction accuracy.

## 1. Introduction

The modernization of the communication infrastructure of the electrical grid, featuring smart sensors, IoT, and edge computing [1], as well as the deregulation of the electric power markets, has enabled the proliferation of distributed generation, mainly from renewable energy sources (RES) [2]. This new paradigm, while actualizing the much sought-after decarbonization of energy generation, has jeopardized the stability of the distribution network due to the intermittency of the aforementioned resources, mainly in isolated power grids, such as non-interconnected islands. The effect of this intermittency is two-fold: From the distribution system operator perspective, uncertainty in RES generation compromises the ability to effectively plan short-term unit commitment scheduling [3,4,5,6], while from the energy market bidder perspective, stochasticity severely constrains bidding strategy and thus, reduces profit margins [7]. An important development in the field of energy transactions is the participation of the energy market in the distribution grid through ancillary services, which is expected to be established in the upcoming years [8].

These shortcomings underline the importance of the application of effective electric load prediction models in the context of multiple operational aspects of the smart grid, such as power stability and security. Especially in the case of micro-grids, storage management is critical and cannot be accomplished without the aid of accurate short-term load forecasts for load shifting and balancing operations [9,10]. Moreover, the grid extension and the increasing exploitation of smart meters affect the efficient operation of the grid, leading to a complex and multifaceted framework [11,12]. As regards the distribution network on the substation level, load forecasting up to one day ahead, could be a valuable asset in the grid’s optimization tasks [13]. Such actions can be carried out, not only by controlling the on-load tap changer (OLTC) and capacitor bank movements, which is currently the industry standard, but also by operation scheduling of batteries in the near future. Load forecasting with multiple time horizons participates in different, interdependent levels of operation of a power grid and thus can make a significant contribution to addressing the aforementioned challenges. A pivotal feature of smart grid is the bidirectional power flow and communication through administrators of generation, transmission, distribution, and end-users. As 8a result, the corresponding energy data contain mixed power-load (hereby referred to as ’mixed load’). While the majority of the load forecasting models found in the literature predict the electric load production or consumption, the differences between them are frequently studied as well. The forecasting of the so-called net load proves useful for tackling load volatility due to increasing RES penetration [14,15,16,17,18]. These net energy load prediction models utilize historic distribution grid load data as well as measurements of weather features on a substation level in order to infer the net active power (AP) demand of the distribution grid.

To this end, the field of computational intelligence, and more specifically, the branch of machine-learning (ML) [19], has proved to be an invaluable source providing a multitude of approaches to solving the aforementioned problem. ML methodologies are capable of extracting knowledge from historical data in order to develop black-box models and avoid the computationally intensive use of first-principle equations. Such algorithms can exhibit a number of important advantages like efficiency, increased prediction accuracy, robustness, etc., but require a number of suitable data to do so. The most common ML methodologies used in the context of load forecasting are mentioned in the following literature review.

Linear regression (LR) belongs to the methods originally used for load forecasting. An approach based on the regression analysis of decomposed time series is presented in [20] for modeling both voltage and electricity demand volatility. A probabilistic approach for handling the uncertainties of power load data is proposed in [21], employing quantile regression, while in [22], weather conditions and electricity prices are also considered. The influence of climatic variables on electricity demand forecasting is examined thoroughly in references [23,24]. The issue of improving the prediction of load consumption data of a set of smart meters is addressed in [25], featuring a periodic autoregressive model with exogenous variables (PARX), which include calendar and temperature information. An important research matter in load forecasting has been the presence of seasonal effects. To this end, different techniques have been proposed, presenting triple exponential smoothing [26], decomposition methods [27,28], or multiple equation time series [29]. Similarly, the maximum weekly load consumption is forecasted for a one-year horizon in [30], where the different components of the decomposed load are modeled by ARIMAX and ARIMA models. These models incorporate previous forecasting errors in the regression equation and thus outperform the simpler AR models in general. The authors of [31] make use of an ARIMA model for electric vehicle charging demand forecasting, the outputs of which are used to formulate a stochastic day-ahead scheduling problem.

Over the last few years, sparsity has emerged as a general principle for signal modeling. Sparse coding refers to the modeling of data signals as the sum of a few basic elements. Although it was primarily used in image processing, recently, there has been considerable interest in electrical signals. The use of sparse coding for modeling and forecasting individual household electricity loads was studied in [32] by implementing the alternating direction method of multipliers (ADMM) algorithm for solving the dictionary learning problem. A number of papers based on sparse Bayesian learning (SBL) have been published during the last decade, featuring weighted SBL [33,34] or combined kernels SBL [35]. More recently, a hierarchical sparsity approach has been proposed [36] for hourly load forecasting, achieving remarkable results and outperforming both well-known sparse techniques and rival linear and non-linear methodologies.

Load forecasting using support vector machines for regression has been gaining popularity in recent years, due to the ability of this method to model the non-linearities present in the electric load prediction problem. The support vector regression (SVR) model in [37] deals with peak load forecasting. A polynomial kernel function is chosen, the parameters of which are optimized through multiple cross-validation. In contrast to conventional SVR models, the idea of pooling information from different multiple individual models with different kernel functions is introduced in [38,39]. The hourly load consumption prediction is attempted in [40], proposing an SVR model which exploits the empirical mode decomposition method to disaggregate a time series into two sets of components, respectively describing the trend and the local oscillations of the energy consumption measurements. A hybrid model [41] has been proposed recently for half-hourly demand forecasting using a modified fire-fly optimization algorithm for tuning the SVR hyperparameters.

Owing to their capability of capturing non-linear correlations, feed-forward neural networks [42] have been extensively applied for the prediction of both load consumption and demand. For the scope of this work, multi-layer perceptron (MLP) and radial basis function (RBF) networks are investigated, and their underlying structure is presented in the next section. The following papers propose the implementation of feed-forward neural networks for hourly load forecasting using historical data on hourly consumption. Particular calendar indices are also used by [43,44], while temperature and humidity data by [45]. These variables are common in most models, as the positive impact of exogenous variables on load forecasting has been confirmed by a number of articles, e.g., [46,47,48,49]. In many cases, the Levenberg–Marquardt algorithm is selected for artificial neural network training showing a better performance over other algorithms [50,51]. According to [52], the proposed multi-layered feed-forward neural network, which is optimized by grey wolf optimizer, demonstrates superior forecasting accuracy compared to simple LR as well as MLP combined with popular metaheuristic methods. An advanced artificial neural network following a novel connection between layers, called the dense average connection, has been proposed recently [53], showing satisfactory results. Aiming to improve the training procedure of a three-layer feed-forward neural network, [54] proposes an advanced backpropagation algorithm. The authors of [55] propose a methodology for short-term bus load forecasting, where an MLP neural network is combined with a bus-clustering algorithm, achieving reduced computational time. A multi-agent system architecture is designed in [56], including the identification of 24-h demand patterns, the classification of days according to these patterns, and finally, a set of MLPs for demand forecasting. Regarding the use of RBF networks, it occurs on a lesser scale. To be more specific, electric load demand forecasting models have been developed featuring short [57] and long-term [58] prediction horizons.

Random forests (RF) constitute an ensemble machine-learning method presenting very good predictive accuracy and have been used in a number of applications, including load forecasting. Several regression models are evaluated in [59], with random forest regressor to provide better short-term load demand predictions than *k*-nearest neighbor regressor and linear regressor in terms of MAPE. A probabilistic load forecasting model based on quantile regression forest is developed in [60] and is enhanced by recursive feature elimination for the purpose of feature selection. Moreover, the authors propose an alternative quantile determination method to alleviate the reliability issue of direct prediction interval construction.

It is apparent from the literature that the problem of electric load forecasting has been addressed by multiple machine-learning methods, but without any of them achieving universal superiority in terms of performance. This observation is confirmed not only by studying the individual research results but also by assessing various benchmark comparisons in the literature [61,62,63]. The inability of universal prediction effectiveness of the aforementioned models is to be expected taking into account the undesirable characteristics of the load forecasting problem, which include non-linearities and high levels of noise in the associated data. Furthermore, load time series are not statistically static [64], due to the volatile, rapidly changing nature of the weather conditions that affect their power generation component. Different classes of machine-learning methods can cope better with some of these issues but usually underperform with respect to others, e.g., linear models are more robust to noise but cannot capture the non-linearities present in the load forecasting problem. To make things worse, though all of these problems are inherent to load forecasting, their mixture composition changes depending on the time horizon one tries to predict for, making it impossible to single out a unique machine-learning method that could outperform the others across different prediction horizons, e.g., linear methods are often found to perform better in short-term horizons, where data tend to be noisy, but the non-linearities can usually be adequately approximated by linear models, but mostly fail in longer time horizons, where the role of the non-linearities is dominant. It should be noted that the previous observation about the inability of a single method to beat all the others is not only tied to the context of load forecasting, but reveals a more generic concept in machine-learning and optimization, as expressed by the “no free lunch” theorem [65]. 

To remedy this predicament, one could resort to using a multi-model approach [66], combining multiple machine-learning methods. Unfortunately, in a real-time deployment scenario, an important practical consideration arises for multi-model schemes: How does one select the most suitable model from a pool of trained models for the next prediction timestep? One solution is to employ a rule-based decision system that uses a priori available knowledge, such as the time of day and measured weather conditions at the substation level. This presents a significant impediment. Not only are the rules of such a system difficult to conceptualize, but they also offer no guarantee of continuously optimal model selection. Doing away with a decision system altogether is also problematic since the individually generated predictions do not offer any actionable insight by themselves. Whether a distribution system operator technician or a RES aggregator, a practitioner requires a single forecast value in order to develop their operations strategy. A practical workaround is to discard such selection rules and instead employ a weighting system that assesses models only by using their past prediction performance [67]. The weighting of the output results of basic forecasting LSTM models in [68] is based on the similarity degree between target and identified standard values of load consumption. Two different approaches for determining the weights of multiple forecasters are followed in [69,70], using a novel incremental ensemble weight updating strategy and the minimum-error method, respectively. Alternatively, an extreme learning machine can be employed for combining the outputs of a pool of forecasts, as in [71]. An intelligent decision-making support scheme, including predictive performance evaluation, model properties analysis, structure and fusion strategy optimization, and optimal model preference selection, is incorporated with an evolutionary ensemble learning method proposed in [72] for short-term load forecasting (STLF) problems. Finally, an automated system is established in [73] based on hidden Markov chains for extracting similar day profiles to obtain the best model from a library of available forecasting models. Differently from the previous works, the output neural network (NN) models result from multiple training cycles based on snapshots [74] or the hidden features of a Random Vector Functional Link network [75]. 

It has become clear that the necessity of providing mixed load forecasts, and indeed for multiple short-term horizons, is a factor of paramount importance in the upcoming transition to smart electricity grids. Moreover, according to the preceding literature review, it is evident that in order to enhance the predictive capability of a model, it should incorporate more than one machine-learning methodologies, which of course should be able to handle the complex dynamic behavior of the mixed load. Finally, such a methodology is necessary to be applicable in an online implementation, which means that the final predictions should be provided in a reasonable amount of time and respond to the behavior of the load through a dynamic decision mechanism. 

Realizing the aforementioned requirements and seeking to fill the corresponding research gaps, in this work, we present a novel forecasting scheme that is able to efficiently address the diverse and adverse characteristics of the load forecasting problem for various prediction horizons. The proposed method seeks to create an ensemble of prediction models based on multiple machine-learning techniques comprising different beneficial characteristics that have only been used individually for load forecasting before. Indeed, the sparse coding method introduced in the proposed model has been published very recently and used for the first time in ensemble schemes. As the participating techniques excel in different aspects of the load forecasting problem, their combined usage introduced in this work provides the ensemble with the ability to outperform each individual method in all the horizons tested. In order to efficiently combine the different machine-learning techniques, the proposed method employs an error-based metric on a rolling window of past predictions. This approach enhances the novelty of the proposed method as it does away with the adversity exhibited by complex, rule-based model selection systems. By combining the beneficial characteristics of the aforementioned techniques, the proposed scheme demonstrates superior performance in terms of prediction accuracy, compared to all the submodels, as well as a recently proposed MLP model ensemble from the literature [76], through a wide range of different prediction horizons, spanning from 15 min to 24 h-ahead. Thus, reliable forecasts can be obtained for: (a) One hour ahead or less, which are valuable for various applications at the transmission and distribution network, (b) one day ahead, contributing to the scheduling of generation sources and (c) intra-day forecasting, so as to achieve better optimization results. As a result, the introduced model ensemble can become a powerful tool for administrators and participants in the energy market, easily exploitable in both operational and managerial tasks of smart grids. It should be noted that, at least to the authors’ best knowledge, no machine-learning approach that is able to handle this range of prediction horizons has been proposed in the literature. Furthermore, the proposed approach expands the existing literature by using mixed power-load data, i.e., data that include renewable generation measurements. Although there is an abundance of work in forecasting the net power load, the literature on mixed-load forecasting is very scarce. It should be pointed out that the employment of mixed measurements is aligned with the requirements of modern smart grids, where the penetration of renewable resources is a key feature.

The paper is structured as follows: Section 2.1 provides a short description of the different ML methods exploited for building the proposed pool of models. In Section 2.2, the proposed approach is presented analytically and then follows the application of the multi-model scheme upon a certain case study in Section 3, where information about the data and the training process and finally results are given in Section 4. Subsequently, in Section 5, the obtained results are discussed and explained. Finally, conclusions and guidelines for future work are outlined in Section 6.

## 2. Materials and Methods

### 2.1. Machine-Learning Methods Short Description

As mentioned earlier, multiple machine-learning methods are involved in the proposed approach. In this subsection, a short description of each one of these methods is provided. Here, we provide a short description of each one of them.

#### 2.1.1. Linear Regression

LR is considered a standard method for addressing problems such as time series prediction, outlier detection, reliability analysis, and feature selection. The regression analysis method is basically a curve-fitting problem. Given a training dataset, $\left(y_{n},x_{n}\right),y_{n}\in R,x_{n}\in R^{l},n=1,2,\ldots ,l$, where $y_{n}\in R$ represents the output or dependent variable and $x_{n}\in R^{l},n=1,2,\ldots ,l$ represents the input vector or regressor [77], the aim is to find a function, f, which fits the data. Subsequently, when an unknown data point $x^{*}$ appears, we can use this function in order to calculate/predict the respective output $y^{*}$ [19]. Equation (1) describes the relation between the input and output variables.

(1) $$y=\theta^{Τ}x+\eta$$

The objective of a regression problem is the estimation of regression coefficients vector θ which arises through the solution of a least squares problem.

(2) $$\hat{\theta }={\left(x^{T}x\right)}^{-1}x^{T}y$$

Although more modern and advanced methods have been developed, LR is still used due to its simplicity and robustness, which are of great importance, especially in online implementation of load forecasting. However, the inability of the method to extract the non-linear behavior of the load is an important disadvantage.

#### 2.1.2. Sparse Coding

When addressing a linear system, for example $x=D\alpha ,x\in R^{m\times n},\alpha \in R^{k\times n}$, the number of predictors, p, can be extremely large. Thus, it is impossible to fit a linear model when p<m, or even when p≈m without overfitting (depending on the noise level), but it may still be possible to fit a sparse linear model that only depends on a reduced number of predictors s, where s<p. The dictionary $D\in R^{m\times k}$ is underdetermined, and therefore, the linear system has infinite possible solutions. The sparse regression (SR) problem is defined as the search for the sparsest solution, i.e., the one with the fewest non-zeros, and is described by Equation (3) [78]:

(3) $${min}_{\alpha \in R^{k\times n}}{\left\|a\right\|}_{0}\;subject\;to\;x=D\alpha$$

where ${\left\|a\right\|}_{0}$ is the $l_{0}$ norm, which counts the non-zero components of a. Although the problem in question is NP-hard, it can often be solved using approximation methods, such as greedy algorithms: Orthogonal matching pursuit [79], thresholding algorithm [80], or relaxation algorithms such as basis pursuit [81] are commonly used for this purpose. SR is a methodology of low complexity that carries the disadvantage of linear correlation assumption between mixed load features, but on the other hand is able to prevent overfitting compared to more complicated ML approaches. A key point stage in the sparse representation procedure is the so-called dictionary learning, which consists of finding the elements of the dictionary (atoms). Dictionary learning can be formulated as a joint unconstrained optimization problem [82], given in the form of (4).

(4) $$\underset{D\in C,a\in R^{\kappa \times n}}{\min}\sum_{i=1}^{n}{\left\|x_{i}-Da_{i}\right\|}_{2}^{2}+\lambda {\left\|\alpha_{i}\right\|}_{0}$$

where $C=\left\{D\in R^{m\times k}s.t.\forall j=1,\ldots ,k,d_{j}^{T}d_{j}\leq 1\right\}$, denotes the feasible space of dictionary D. The role of the parameter λ is to regulate the sparsity level of the coefficient vector. 

Dictionary learning can be accomplished using several algorithms, like online dictionary learning [83], method of directions [84], K-SVD [85], stochastic gradient descent [86], or LASSO [83].

#### 2.1.3. Support Vector Regression

The next method to be included in the pool of models constitutes an extension of support vector machines in regression and is called SVR. The basic idea of this methodology is the use of a non-linear transformation $\varphi \left(\cdot \right):R^{n}\to R^{n_{h}}$ that maps the real data into a multi-dimensional space [87] and the subsequent application of LR. According to this approach, a linear function s is supposed to exist in the multi-dimensional space, which models the non-linear relation between the input and output data of the initial space [88]. Such an equation is given in (5).

(5) $$s\left(x\right)=w^{T}\varphi \left(x\right)+b$$

where $\varphi \left(x\right)$ denotes the kernel function and $w^{T}\in R^{n_{h}}$, b∈R are the regression coefficients. The problem of calculating the variables w and b is reduced to the minimization of the structural risk functional.

(6) $$R=\underset{w}{\min}\frac{1}{2}{\left\|w\right\|}_{2}^{2}+C\sum_{i=1}^{n}{\left|s\left(x_{i}\right)-y_{i}\right|}_{\epsilon }$$

where y contains the real measurements and C is a penalty term used to balance between data fitting and overfitting.

The employment of the kernel trick allows SVR to acknowledge the presence of non-linearity in mixed load series. However, when a separating hyperplane in a given dimension cannot be found, then it is required to move in a higher dimension. In this case, the computational cost will increase as well. Furthermore, the use of support vectors makes the method sensitive to noisy data and outliers.

#### 2.1.4. Neural Networks

**Figure 1:**
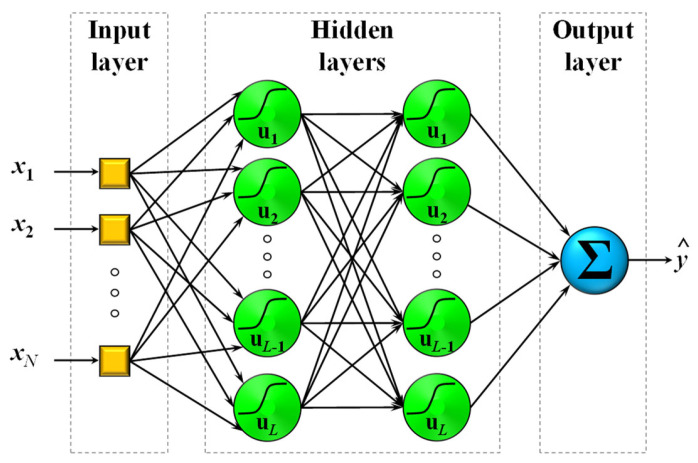
A typical fully connected multi-layer perceptron (MLP) neural network (NN) structure comprising of N inputs, $x_{1},\ldots ,x_{n}$, 2 hidden layers of L neurons each, and one-dimensional output, $\hat{y}$.

Neural networks (NNs) constitute an important family of black-box modeling techniques. NNs are very accurate, robust, fault-tolerant, and flexible to adapt to any process given a suitable number of quality data. The proposed model ensemble includes two representative NN architectures, namely the MLP and the RBF. MLPs identify the process dynamics and form the model by guiding the input data through weighted successive layers of non-linear functions (threshold, sigmoid, etc.) called activation functions or nodes. The input (activity) $\mu_{l}\left(x\right)$ to each one of the L nodes is the weighted sum of all N input variables to that node.

(7) $$\mu_{l}\left(x\right)=x\cdot w$$

where w are the weights corresponding to each variable of the input vector x. The intermediate layers between the input and the output are called hidden layers. The schematic of a typical MLP NN with 2 hidden layers is presented in Figure 1. The prediction produced by an MLP is the weighted sum of the final layer outputs. Due to the existence of non-linear characteristics in mixed-power load data, MLPs certainly seem like a promising method for the problem at hand.

On the downside, MLP training is usually performed by some form of backpropagation technique which usually requires more than one intertwined iterative procedure to fully optimize the involved parameters, i.e., the number of layers and nodes, the weights, etc. Depending on the size and architecture of the MLP network, and the input space, this procedure may become computationally intensive, and thus it is commonly performed offline. A quite critical drawback of all backpropagation-based techniques is that they get easily trapped in local minima. In this case, the provided solution may not be satisfactory, a fact which leads to a tedious retraining procedure.

**Figure 2:**
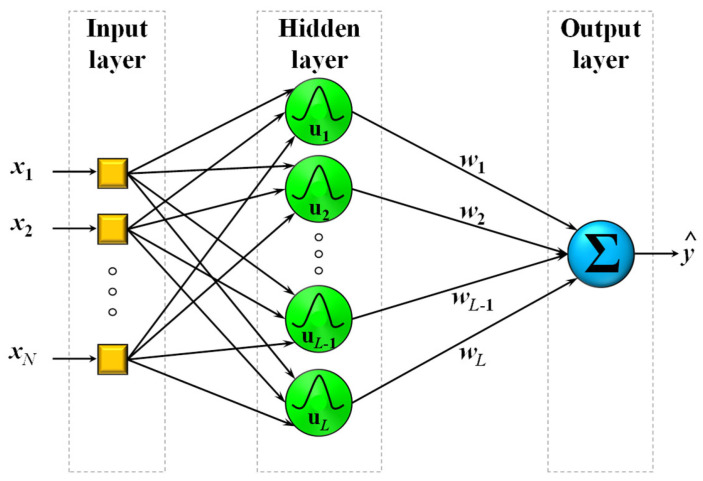
A typical Gaussian-based radial basis function (RBF) neural network (NN) structure comprising of N inputs, $x_{1},\ldots ,x_{n}$, L neurons in the hidden layer, and one-dimensional output, $\hat{y}$.

RBFs are similar to the MLPs in the sense that data are fed through the input layer and follow a straight path to the output layer but they differ in that there exists only one hidden layer which comprises radially symmetric activation functions (Gaussian, quadratic, thin plate spline, etc.). A typical RBF NN using Gaussian activation functions can be seen in Figure 2. The input layer distributes the data of the N inputs to the L nodes of the hidden layer, which are positioned to a specific point of the input space through a process of training. The activity $\mu_{l}\left(u_{k}\right)$ of each node is calculated using the Euclidian distance between the input data $u_{k}$ and the center $c_{l}$ of each node.

(8) $$\mu_{l}\left(u_{k}\right)={\left\|u_{k}-c_{l}\right\|}_{2}=\sqrt{\sum_{i=1}^{N}{\left(u_{i,k}-c_{i,l}\right)}^{2}},l=1,2\ldots ,L,k=1,2,\ldots ,K$$

where K is the number of training data. The chosen RBF $g_{k}$ receives the activity value and calculates the node output. The linear combination of all hidden layer node outputs provides the NNs prediction ${\hat{y}}_{k}$

(9) $${\hat{y}}_{k}=g_{k}\cdot w$$

The training algorithm for an RBF NN is usually broken down into two phases, the first of which discovers the optimal number and location of the hidden node centers in the input space, while the second one calculates the weights w usually through simple LR. Due to the fact that the training process is broken into two phases, RBF NNs are able to use very fast algorithms. In fact, some of the current RBF training techniques [89] are deterministic and non-iterative, requiring only a single pass of the data to converge, in contrast to other NN architectures which (a) are epoch-based requiring multiple passes of the data and (b) are stochastic requiring multiple runs to overcome their sensitivity to initial conditions. RBF networks provide very strong interpolation tools, usually outperforming other NN-based techniques provided that dense and good quality training data are available. In the absence of adequate training data though, their performance may become rather poor. Therefore, their application, in combination with the other models of the pool, can make a significant contribution to mixed-power load prediction.

#### 2.1.5. Random Forests

**Figure 3:**
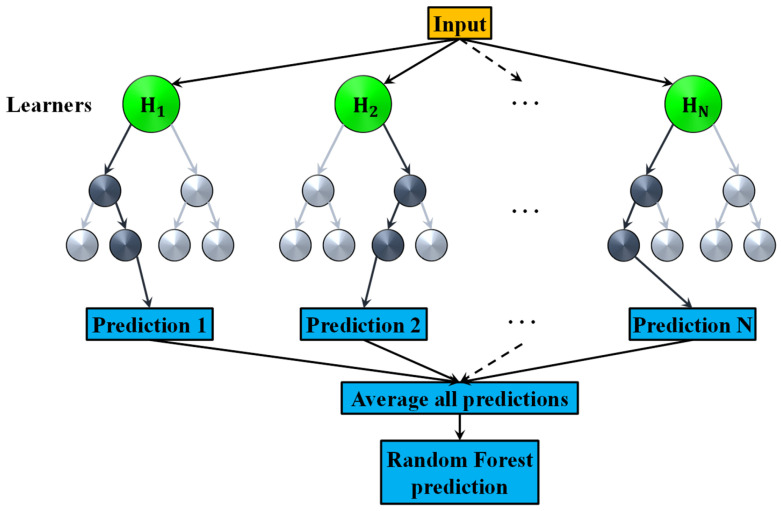
A typical random forest architecture comprising of N tree learners, $H_{1},H_{2},\ldots ,H_{N}$. The prediction set for each learner is averaged to produce the final predictions.

The last method involved in the proposed approach is RF [90]. As the name suggests, an RF is a tree-based ensemble, with each tree depending on a collection of random variables. More formally, for a p-dimensional random vector $X={\left(X_{1},\ldots ,X_{p}\right)}^{T}$ representing the real-valued input or predictor variables and a random variable Y representing the real-valued response, we assume an unknown joint distribution $P_{XY}\left(X,Y\right)$. The goal is to find a prediction function $f\left(X\right)$ for predicting Y. The prediction function is determined by a loss function $L\left(Y,f\left(X\right)\right)$ and defined to minimize the expected value of the loss

(10) $$E_{XY}\left(L\left(Y,f\left(X\right)\right)\right)$$

where the subscripts denote expectation with respect to the joint distribution of X and Y. Intuitively, $L\left(Y,f\left(X\right)\right)$ is a measure of how close $f\left(X\right)$ is to Y. It penalizes values of $f\left(X\right)$ that are a long way from Y. As for simple LR, squared error loss could be a typical choice of L. Ensembles construct f in terms of a collection of linear estimators of x, the so-called “base learners” $h_{1}\left(x\right),h_{2}\left(x\right),\ldots ,h_{J}\left(x\right)$, where J denotes the number of trees and is user-specified, according to the following iterative procedure. Let $D=\left\{\left(x_{1},x_{1}\right),\ldots ,\left(x_{N},x_{N}\right)\right\}$ denote the training data, with $x_{i}={\left(x_{i,1},\ldots ,x_{i,p}\right)}^{T},i=1,\ldots N$. For each $j\in \left(1,J\right)$, a bootstrap sample $D_{j}$ of size N is extracted from D and a corresponding tree $h_{j}\left(X,\Theta_{j}\right)$ is derived implementing the binary recursive partitioning [91]. The prediction extraction using a standard RF regressor is depicted in Figure 3. For each unsplit node of the tree, the best binary split among all binary splits on the $m\in \left(1,p\right)$ predictors, is found. The component $\Theta_{j}$ is used to inject randomness first by bootstrap sampling and second by the random subset of m predictors. Once the base learners are found, a prediction at a new point x is given by

(11) $$f\left(x\right)=\frac{1}{J}\sum_{j=1}^{J}h_{j}\left(x\right)$$

RF is a simple and reliable forecasting tool. Its main limitation is the trade-off between performance and the number of trees. Increasing this parameter can lead to more accurate predictions and prevent overfitting but can also make the algorithm too slow and ineffective for real-time predictions. It is, therefore, understandable that this methodology may prove suitable in specific areas of the dataset.

### 2.2. Machine-Learning Model Ensemble

Recognizing the individual advantages and disadvantages of the machine-learning methods described in Section 2.1, the proposed scheme seeks to create an ensemble that will successfully combine their merits in a single approach. For example, neural-network-based models such as RBF do exhibit superior prediction performance only as long as the input data point lies well within the domain of the input training dataset, otherwise it fails. On the other hand, linear and sparse prediction models, in general, show much better extrapolative performance, even though they are unable to capture more complex, non-linear dynamics. In other words, by toggling between the robust linear models and the more sensitive but also more effective non-linear ones, a superior approach to load time series prediction can be constructed.

In order to obtain the best possible performance of each sub-model, their optimal training configuration has to be determined. Starting with the simpler methods used, a linear and an SR model are trained by least squares and fast iterative shrinkage thresholding algorithm, respectively, the latter being a faster implementation of the corresponding iterative shrinkage thresholding algorithm used for load forecasting [36]. In the case of the sparse coding approach, sparsity is induced by the $l_{2}$ norm and the regularization parameter was set by trial and error to 0.01. Subsequently, a random forest regressor is employed, where the number of decision trees is selected to be 15 so as to keep the training time at a reasonable level without reducing its predictive ability. As regards the non-linear methods, an SVR model with Gaussian kernel function was developed [92], using sequential minimal optimization for training and Bayesian optimization to optimize the model’s hyperparameters [93]. Two NN models are also introduced, featuring two different architectures. The first one is a two-layered MLP network trained by the Levenberg–Marquardt backpropagation algorithm [94], following a 10-fold cross-validation. The neurons of each layer are chosen by trial and error as 20 and 10. It is noted that, in order to compensate for the performance dependence of the MLP training methods to initialization, the training procedure was conducted 10 different times, with different randomly initialized weights of the network. The second NN uses an RBF architecture and is trained using the fuzzy means technique [95], an algorithm that has found many successful applications due to the increased accuracy it provides [96,97,98] combined with fast training times [99]. In this work, the FM algorithm has been tested for a range of fuzzy sets between 4 and 15. When deployed online, the proposed approach evaluates a MAE metric on a rolling window of past predictions coming from a pool of trained models in order to create a weight vector for the next timestep prediction. An important item of the proposed method to be specified is the length of the rolling window. It can be easily inferred that this depends not only on the prediction horizon but also on the statistical properties of the predicted variable (a more volatile, non-stationary time series would require shorter rolling window horizons). Once the model pool has been populated by trained models, the optimum length of the rolling window is calculated in an exhaustive search manner over the same validation data in the range of 3–15 regressive timesteps. The proposed method operates as follows: For each timestep *k*, all trained models in the pool are evaluated concurrently. Their current prediction performance is assessed by applying the MAE metric on their previous predictions up to a rolling time window of length $h_{w}$

(12) $${MAE}_{i}(k)=\frac{\sum_{j=0}^{h_{w}-1}\left|{\hat{y}}_{i}\left(k-j\right)-y(k-j)\right|}{h_{w}}$$

where ${\hat{y}}_{i}(k)$ are the predictions of the *i*-th model and y are the actual values of the times eries at timestep *k*. Then, the MAE metric is used to calculate the prediction weight of each model for the next timestep *k +* 1.

(13) $$w_{i}(k+1)=\frac{{MAE}_{i}^{-1}(k)}{\sum_{i=1}^{N}{MAE}_{i}^{-1}(k)}$$

where ${MAE}_{i}$ is the MAE of the *i*-th prediction model, *N* is the total number of models in the model pool, and $w_{i}$ is the prediction weight for the next timestep. The prediction output of the proposed method is calculated as the weighted sum of the model predictions ${\hat{y}}_{i}$

(14) $$\hat{y}\left(k+1\right)=\sum_{i=1}^{N}w_{i}(k+1){\hat{y}}_{i}(k+1)$$

**Figure 4:**
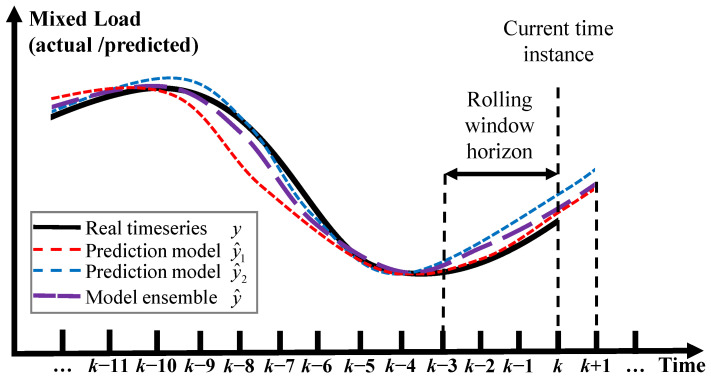
Schematic for a two-model version of the proposed method, where y denotes the real load, ${\hat{y}}_{i}$ the prediction of the *i*-th model, $\hat{y}$ the weighted prediction and *k* the current timestep. The ensemble model recognizing the superiority of ${\hat{y}}_{1}$ over ${\hat{y}}_{2}$, within the rolling window adapts its weights accordingly, achieving highly accurate prediction for the next timestep *k +* 1.

A snapshot of a two-model example version of the proposed method is shown in Figure 4. Note that the proposed method combines the strengths of the individual models by placing greater weight on the current better-performing model for the time window of length *h_w_*. At first, both ${\hat{y}}_{1}$ and ${\hat{y}}_{2}$ models appear ineffective as individual predictors of the y time series. However, after closer inspection, ${\hat{y}}_{2}$ performs better for the first half of y, while ${\hat{y}}_{1}$ for the second half. By placing greater weight on the model with the best past prediction performance within the horizon *h_w_*, the proposed method is able to toggle towards the best available model for the current circumstance. The result is an overall superior prediction performance.

## 3. Case Study

### 3.1. Problem and Data Description

The main goal of this paper is to develop a methodology in order to implement a load forecasting tool able to provide accurate mixed load predictions over several different time horizons and in particular 15 min, 1-h, 2-h, 3-h, 6-h, and 24-h. This case study makes use of real data from a high voltage/medium voltage substation located in mainland Europe, measured during the years 2017–2018. The MV distribution network contains multiple photovoltaic sites. As a result, the data measurements in question constitute mixed power-load recordings, which correspond to the mixed AP demand of the distribution grid from the transmission grid. The load measurements have been recorded every minute and contain the mixed AP demand, as well as cloud coverage, wind speed, humidity, and temperature, as measured from the substation’s weather station. Due to practical concerns, individual power generation or weather data from the aforementioned photovoltaic sites should not be taken into account for the creation of the input dataset since these will normally not be available for a real-life implementation. In short, in this work, we rely on the substation’s historical measurements of load and weather conditions in order to create a prediction model of the mixed AP demand of the grid.

### 3.2. Data Preprocessing and Model Training

**Figure 5:**
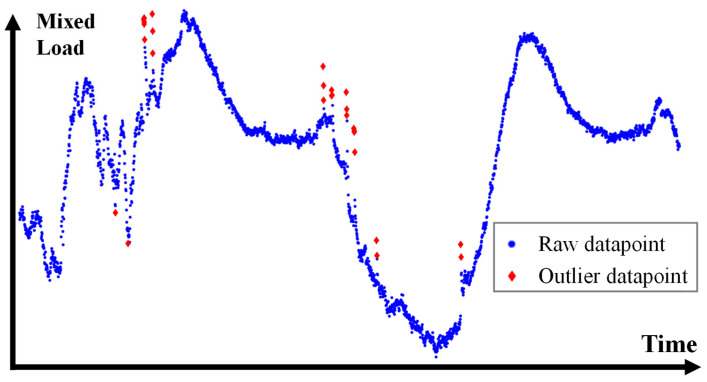
Operation of the rolling median threshold outlier detection algorithm. The data points marked as outliers exceed the median value of the time window multiplied by a user-specified threshold factor.

Unavoidably, the substation measurements contain large periods of missing or corrupt data owing to sensor downtime or malfunction. For the scope of this case study, no missing data imputation has been performed-instead, corrupted data and outlier removal was the main focus of the preprocessing operation. Due to the sheer size of the dataset, manual preprocessing was impossible, mandating the creation of a bad data detection routine. Corrupted values were decidedly easy to detect since the corresponding AP signal exhibited unusually low variance around a constant value. However, outlier values on mixed load data were a challenge to successfully handle—a review of the challenges of this topic, as well as effective techniques, is available on [100]. The chosen technique must be sufficiently effective at classifying outliers in data, while avoiding false positives. In this case study, a rolling median window threshold approach is used, as it was found to compromise well between the aforementioned points. A two-day snapshot from the application of this algorithm to raw electrical load data is presented in Figure 5. The outliers usually originate from noisy sensor readings [101]. As part of data preprocessing, a resampling step also took place, where each sample was defined as an average of 15 one-minute measurements.

The task of input variable selection is closely related to the prediction horizon. All models developed in the context of this study are considered autoregressive with exogenous variables, as they use inputs that consist of previous values of the output and weather data. A set of inputs was initially constructed for each prediction horizon based on the literature. Subsequently, the contribution of these variables to the prediction accuracy improvement was examined by trial and error, sometimes leading to shorter input sets for some of the horizons. Alternatively, other approaches, such as gradient boosting decision tree and Pearson correlation coefficient [102], attention mechanism [103], or Exploratory Data Analysis [104], are considered to have an effective contribution during input features reduction and selection. However, it is important to note that for each horizon, inputs remain the same for all machine-learning methods used in the present study.

The selected input variables which all models accept could be divided into 4 categories, as described in Table 1, namely (a) current and past AP values, (b) difference between current and past AP values, (c) average of past AP values, and (d) weather measurements. It has to be noted that $p^{\left(t\right)}$ values contain the current and past, average and difference measures of the AP values, ${\hat{p}}^{\left(t+s\right)}$ is the output, i.e., the mixed power load *s* fifteen-minute intervals ahead, whereas $w^{\left(t\right)}$ components contain the respective weather-related inputs of cloud coverage, wind speed, humidity, and temperature, respectively.

The choice of the particular set of input variables can be justified as follows: The fact that electric load time series presents a strong dependency on previous values [47] strengthens the selection of such input variables in the form of (a). Trying to capture the trend of electrical load, differences (b) between current and previous AP values are frequently employed [31]. The implementation of past value averages (c) is also quite important, according to the literature [48]. Finally, the introduction of weather data (d) is undoubtedly an improving factor in the predictions [23,24,105]. At this point, it is important to mention that during the training stage of the forecasting model, the weather inputs $w^{\left(t\right)}$ are introduced as measured values of actual weather data acquired at the *t* time index. On the contrary, in an online implementation of the model, future weather data $w^{\left(t+s\right)}$ will be unknown and replaced by weather predictions, therefore introducing additional uncertainty.

**Figure 6:**
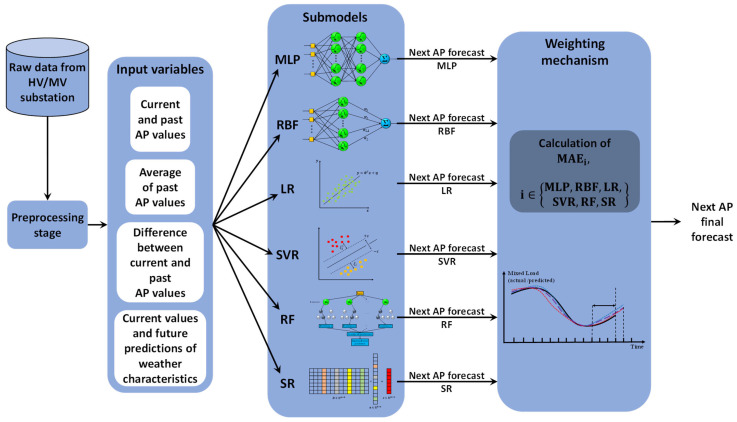
Overview of the proposed model ensemble. Its application in mixed load forecasting comprises a series of steps, i.e. raw data acquisition, data preprocessing, collection of input variables, splitting of the dataset in a training and a testing subset, training of submodels, generation of the next AP forecast by each submodel, weighting of the individual predictions, and, lastly, calculation of the next AP final forecast.

Once the preprocessing stage has been completed and input variables have been selected, the dataset was partitioned in a yearly manner in order to select the training datasets. At this point, an important consideration should be made. As mentioned in the introductory section, the load time series consists of a load and generation component. The statistical properties of both of these components are not static in relation to time, especially on a long-term scale. The network physically expands, incorporating more consumers as well as RES generators, each with different load and generation profiles, respectively. Therefore, it makes sense to select training datasets as close to the actual prediction interval as possible. Since the available data concern two successive years, the data corresponding to 2017 were selected as the training subset, and the data corresponding to 2018 were selected as the testing dataset. A point worth mentioning is that no permutation step is taking place before training. This means that the data used for testing are considered completely unseen for the proposed model, yielding a more reliable forecasting model. Due to confidentiality reasons, the real and predicted mixed load values have been normalized in order to be presented. Finally, it should be noted that models that require a validation step during training, namely models based on MLP and RBF NNs, do so using 10-fold cross-validation, while in the case of models that require multiple training runs for each training seed (see MLP), the best-performing model on the validation data is kept. An overview of the implementation of the proposed model is provided in Figure 6, which illustrates, in the form of a block diagram, the entire sequence of steps that take place, starting from the acquisition of the raw AP data from the substation to the derivation of the final forecasts. It has to be highlighted that this figure is generic and does not refer to a particular prediction horizon. 

At this point, it should be mentioned that in order to evaluate the accuracy of the proposed method, it was considered appropriate to compare it with a model ensemble from the literature. To be more specific, we employed a method proposed for load forecasting based on an ensemble of multiple MLP neural networks [76]. Consequently, following the experimental protocol described in this work, a number of feed-forward NNs, with a single hidden layer, were trained on 14 different random initializations of the weights. For each initialization, the number of neurons in the hidden layer ranged from 3 to 50. The hyperbolic tangent sigmoid function was selected as the transfer function among the NNs’ layers, while all NNs were trained using the resilient backpropagation algorithm. The neural networks were arranged in ascending order with respect to the MAPE error on a common validation set, which, in this case, was defined as 20% of the training dataset. Then, the networks corresponding to the first 5 MAPE errors were selected, and the final forecasts were obtained by averaging the individual forecasts of these 5 models.

## 4. Results

**Figure 7:**
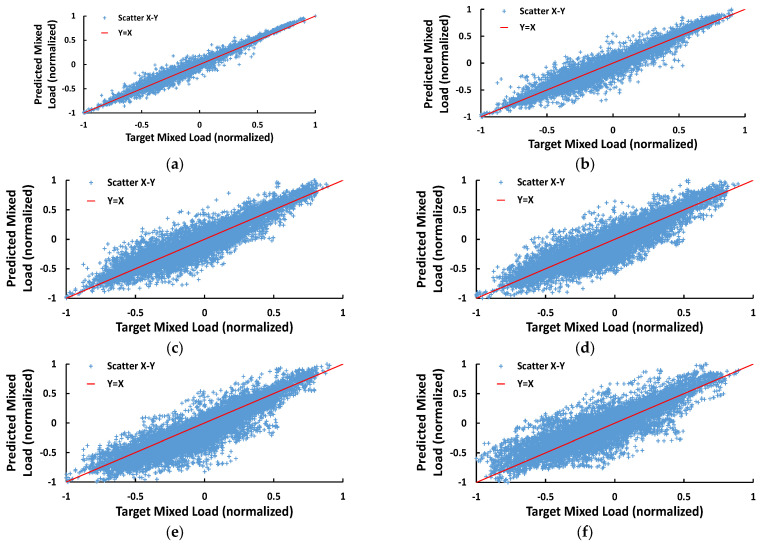
Scatterplots of actual versus predicted mixed load for (**a**) 15-min, (**b**) 1-h, (**c**) 2-h, (**d**) 3-h, (**e**) 6-h, and (**f**) 24-h ahead prediction. The predicted values residing on the diagonal line are identical to the actual values. Each mark refers to a data point and shows the deviation of its predicted value from its actual value.

In this section, the results of extensive simulations of the proposed model are presented. A set of scatterplots is shown in Figure 7a–f, representing the actual versus the predicted values mixed load values for 1, 2, 3, 6, and 24-h-ahead horizons, respectively, through the whole testing dataset. The diagonal line implies a complete match between real values and forecasts. The axes are presented in units of normalized AP.

Additional results are provided in Table 2, which contains information about the forecasting performance of the proposed method in comparison to the individual machine-learning methods comprising the model pool. In order to distinguish the results for different prediction time horizons, the table is divided into sections. The accuracy of model predictions is evaluated through the correlation coefficient (R^2^), RMSE and MAE, considering them as representative and efficient criteria [106]. For comparative reasons, the table also contains the values of the indices for all submodels, as well as their percentage of ranking in the first place. This quantity, labeled as “Rank 1” in Table 2, denotes how many times each submodel scored the 1st rank among all submodels, i.e., achieved the lowest MAE.

**Figure 8:**
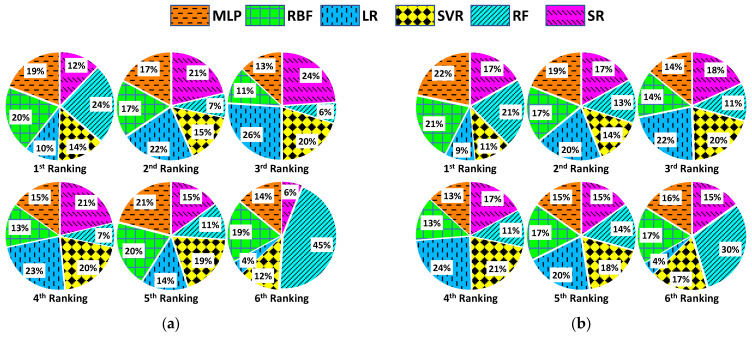

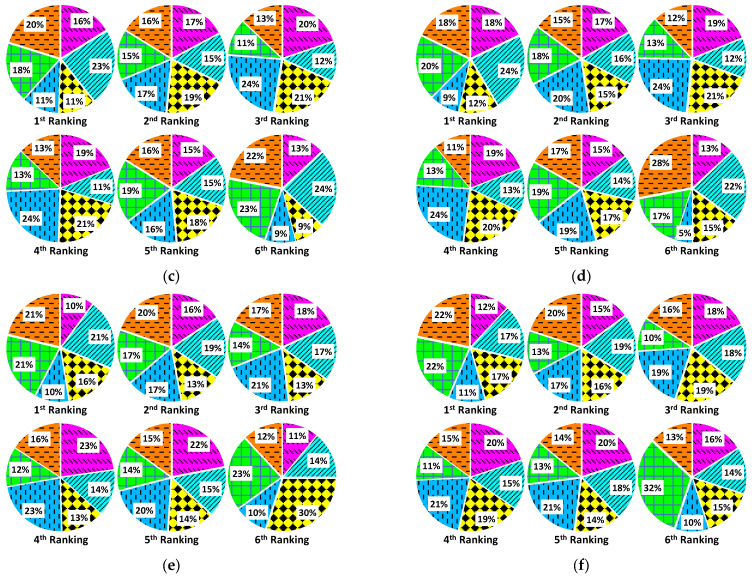
Pie charts depicting the ranking of the submodels included in the proposed model ensemble for (**a**) 15-min, (**b**) 1-h, (**c**) 2-h, (**d**) 3-h, (**e**) 6-h, and (**f**) 24-h ahead prediction. Each pie chart refers to a ranking position and shows the percentage that each submodel was ranked in that position. Each submodel is represented by a different color and pattern.

**Figure 9:**
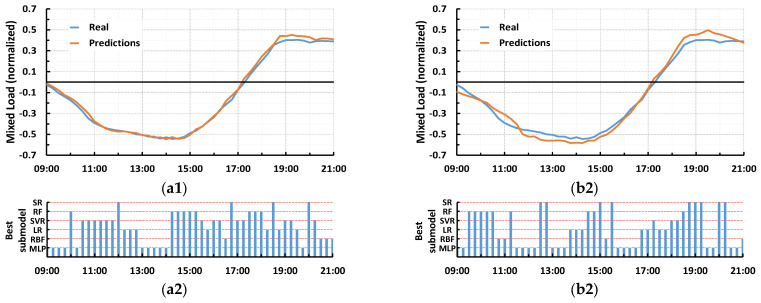

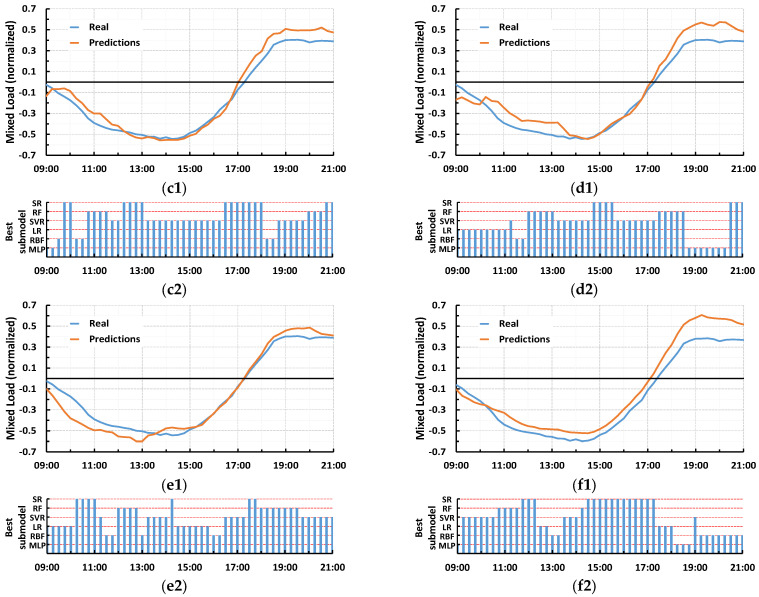
Results for a randomly selected 12-h window for (**a**) 15-min, (**b**) 1-h, (**c**) 2-h, (**d**) 3-h, (**e**) 6-h, and (**f**) 24-h ahead predictions. Subgraphs labeled 1 depict actual and predicted value results, whereas subgraphs labeled 2 depict the best submodel performance results.

The aforementioned form of ranking of the submodels can be seen graphically in Figure 8. More specifically, each one of Figure 8a–f refers to 15 min, 1, 2, 3, 6, and 24-h prediction horizons, respectively. Each one of these subfigures contains 6 pie charts, denoting 1st to 6th rank for the models. To be more specific, each pie chart shows the percentages corresponding to how many times each submodel ranked in the respective place, according to its weighted MAE. For example, the 2nd pie of Figure 8a implies that for 15 min-ahead forecasting, the MLP submodel ranked in the 2nd place among all models with a percentage of 17%, the SR submodel with a percentage of 21%, etc. Finally, analytical graphs are provided for each prediction time horizon, with Figure 9a1–f1 to depict forecasts of 15 min, 1, 2, 3, 6, and 24 h-ahead, respectively, where a randomly chosen 12-h time window (from 09:00 to 21:00) of real AP values and the respective predictions are shown for an arbitrarily chosen day belonging to the testing subset (the same day and the same window is used for all horizons). These graphs are accompanied by Figure 9a2–f2, which indicates which submodel has the largest weight for every predicted data point using a bar plot.

## 5. Discussion

In the context of the case study, multiple experiments were conducted, and the results are explained and discussed here. At this point, we should point out that providing accurate predictions is indeed a challenging task due to both grid and data-related reasons. First, the system’s expandability can be a limiting factor for the accuracy of future forecasts. At the same time, this is reinforced by inherent characteristics of the load time series, such as non-linearity and uncertainty. In the face of these challenges, the proposed method seems to be quite effective, providing reliable predictions. From Figure 7a–f, we can draw conclusions about the quality of predictions. When the prediction time horizon is too short (Figure 7a), the forecast error is distributed close to the diagonal line, which implies quite accurate predictions. While we are trying to increase the prediction horizon, the forecasts are getting less accurate (Figure 7b–f), as obviously, the pairs of real and predicted values are scattered further from the ideal line.

Looking at Table 2, we observe that the proposed model outmatches all individual submodels, and the competitive MLP model ensemble in terms of MAE, and R^2^ and RMSE. Moreover, this conclusion applies to all prediction time horizons. As the prediction horizon gets longer, the forecasting error increases, which is absolutely reasonable. The only exceptions are the R^2^ and RMSE values obtained by the MLP model ensemble for 2 h prediction horizon, which slightly exceeds those of the proposed model. However, these differences cannot be considered significant as they are marginal, while on the other hand, the corresponding value of the MAE index clearly favors the proposed method. A result worth mentioning is the improvement of the multi-model performance over the current best sub-model that occurs in most cases while the horizon is getting longer. More specifically, the reduction of MAE that the proposed approach achieves over the best of the individual models ranges from 0.03411 to 0.3156. Such an improvement in performance could be partly explained by the occurrence of uncertainty in the load time series. As the prediction horizon is getting longer, the level of uncertainty is also increased, which is better addressed by the ensemble model than each individual submodel alone. 

Regarding the efficiency of the individual models of the pool, the results of MAE, RMSE, and R^2^ show that there is not just one model to prevail over the others in all cases. For the shorter prediction horizons and, more specifically, up to 3 h, LR and SR appear to achieve marginally smaller forecasting errors than their non-linear counterparts. Although the non-linearities are an intrinsic characteristic of mixed load [107], this behavior becomes more apparent as the prediction horizon is getting longer. As a result, models which are based on LR are able to provide robust results for very short-term forecasts. On the other hand, one major advantage of neural networks is their capability of modelling non-linear systems. An important observation is that neural networks appear to perform better for longer prediction horizons, and this can be attributed to the fact that, as the prediction horizon is getting longer, the non-linear properties of the load are becoming more dominant. Therefore, when predictions for longer horizons are required, MLP neural networks take the lead. However, the same does not apply to RBF networks. As stated above, in order for RBF networks to perform well, dense and suitable data are required. Consequently, their performance is reduced for 24-h prediction horizons, where the input information is poorer due to the resampling process. Although the remaining models of the pool, SVR and RF networks, present a moderate predictive capability, they contribute positively to the overall performance of the proposed model. This conclusion confirms our claim of the need to use multiple models in order to enhance the reliability of load predictions.

Looking at the results of actual and predicted values in Figure 9 we confirm that, as the time horizon increases, accurate load forecasting becomes more and more difficult. Continuing with the subfigures of Figure 9 that show the alternation between submodels in order to maintain the accuracy of predictions, we conclude that the weighting mechanism of the proposed model seems to perform adequately regardless of the time horizon. It can easily be seen that quite reliable forecasts are obtained during the steady rise or fall of the actual AP values. On the contrary, predictions become less accurate when the AP presents great fluctuation.

Several quite interesting conclusions can also be drawn from the pie charts in Figure 8. Each percentage in the pies represents the degree to which the respective model yielded the highest weight or equivalently the lowest MAE. The highest percentages of the first rank (above 18%) belong to MLP, RBF, and RF, and this applies for all horizons except that of 24 h, where SVR takes the place of RF. RF, in particular, scores lower MAE most of the time when the prediction horizon does not exceed 3 h. Beyond that point, RBF neural networks outperform the rest of the submodels. An interesting observation is that the aforementioned models have equally high percentages in the sixth rank. Thus, these methods either achieve very good or poor performance. This observation is quite significant and strongly enhances the usefulness and effectiveness of our proposed method. The percentages of the rest of the pool models are, in most cases, divided into the intermediate rankings, with the exception of the high percentage of SVR in the sixth rank for the 6-h horizon.

## 6. Conclusions and Prospects

Achieving reliable electric load forecasts is of paramount importance for the smooth operation of electric power grids. However, the intrinsic volatility of the electric load makes its prediction particularly hard. Therefore, we assume that the load behavior is influenced by multiple input variables, which differ depending on the data to be predicted.

The mixed load forecasting task has been addressed by a variety of machine-learning methods, and it has been observed that none is able to provide equally accurate results for any testing dataset. In the present study, a mixed power-load forecasting model is introduced, which employs the predictions coming from several individual models, namely MLP and RBF neural networks, LR, SVR, RF, and SR. These forecasts are weighted based on how accurate they have been and then added to calculate the final forecast value. The proposed model provides predictions for different time horizons, spanning from 15 min to 24 h. The extended results presented using real data sensed from a high voltage/medium voltage substation show the superiority of this novel approach compared to all the individual models as well as an MLP model ensemble for every prediction horizon tested. Thus, the proposed multi-model forecasting scheme constitutes a powerful method capable of greatly enhancing the operation of the modern electricity grid, with potential practical applications in network planning, operation, and management.

It should be noted here that a limitation of the present study is that it did not involve predictions for long-term horizons. Although investigating longer prediction horizons is outside the scope of this work, we believe the proposed model ensemble could serve as the basis for designing such a tool. On the other hand, it is quite probable that a different set of input variables, presenting higher correlation with the long-term evolution of the mixed load would be needed in this case.

Driven by the remarkable performance of the proposed methodology in mixed load forecasting, its application could be extended to other critical sectors of the smart grid, such as forecasting the electricity price and the production from RES, in order to more efficiently schedule conventional sources. A fruitful application would also be to forecast the residential demand or the aggregated load corresponding to several substations. Another promising direction for future research towards this direction includes the integration of Graph Neural Networks, which have been proved to be a promising candidate due to their ability to successfully interpret spatiotemporal features of the input data.

## Figures and Tables

**Table 1:** Description of training variables of the forecasting models for the different prediction horizons examined in the case study. Each row of the table refers to the different groups of input variables, whereas the last row refers to the output variable.

Prediction Horizon	15 min $\left(t+1\right)$	1 h $\left(t+4\right)$	2 h $\left(t+4\right)$	3 h $\left(t+12\right)$	6 h $\left(t+24\right)$	24 h $\left(t+96\right)$
Current and past AP measures	$\begin{array}{c} p^{\left(t-i\right)},\\ i=0,95,671 \end{array}$	$\begin{array}{c} p^{\left(t-i\right)},\\ i=0,4,92,668 \end{array}$	$\begin{array}{c} p^{\left(t-i\right)},\\ i=0,8,88,664 \end{array}$	$\begin{array}{c} p^{\left(t-i\right)},\\ i=0,12,84,660 \end{array}$	$\begin{array}{c} p^{\left(t-i\right)},\\ i=0,24,72,648 \end{array}$	$\begin{array}{c} p^{\left(t-i\right)},\\ i=0,96,576 \end{array}$
Average AP measures	$\frac{\sum_{n=0}^{3}p^{\left(t-n\right)}}{4}$	$\frac{\sum_{n=0}^{3}p^{\left(t-n\right)}}{4}$	$\frac{\sum_{n=0}^{7}p^{\left(t-n\right)}}{8}$	$\frac{\sum_{n=0}^{11}p^{\left(t-n\right)}}{12}$	$\frac{\sum_{n=0}^{23}p^{\left(t-n\right)}}{24}$	$\frac{\sum_{n=0}^{95}p^{\left(t-n\right)}}{96}$
Difference AP measures	$\begin{array}{c} p^{\left(t\right)}-p^{\left(t-i\right)},\\ i=1 \end{array}$	$\begin{array}{c} p^{\left(t\right)}-p^{\left(t-i\right)},\\ i=4 \end{array}$	$\begin{array}{c} p^{\left(t\right)}-p^{\left(t-i\right)},\\ i=8 \end{array}$	$\begin{array}{c} p^{\left(t\right)}-p^{\left(t-i\right)},\\ i=12 \end{array}$	$\begin{array}{c} p^{\left(t\right)}-p^{\left(t-i\right)},\\ i=24 \end{array}$	$\begin{array}{c} p^{\left(t\right)}-p^{\left(t-i\right)},\\ i=96 \end{array}$
Weather measures	$\begin{array}{c} w^{\left(t+i\right)},\\ i=0 \end{array}$	$\begin{array}{c} w^{\left(t+i\right)},\\ i=4 \end{array}$	$\begin{array}{c} w^{\left(t+i\right)},\\ i=4,8 \end{array}$	$\begin{array}{c} w^{\left(t+i\right)},\\ i=4,8,12 \end{array}$	$\begin{array}{c} w^{\left(t+i\right)},\\ i=16,20,24 \end{array}$	$\begin{array}{c} w^{\left(t+i\right)},\\ i=88,92,96 \end{array}$
Future AP forecasts (output variable)	${\hat{p}}^{\left(t+1\right)}$	${\hat{p}}^{\left(t+4\right)}$	${\hat{p}}^{\left(t+8\right)}$	${\hat{p}}^{\left(t+12\right)}$	${\hat{p}}^{\left(t+24\right)}$	${\hat{p}}^{\left(t+96\right)}$

**Table 2:** Performance of the proposed multi-model scheme, the MLP model ensemble of [76], and individual machine-learning models for each prediction horizon. The values of ΜAΕ, RMSE, and R^2^, achieved by each model, are presented, as well as the percentage that each submodel achieved the lowest MAE among all submodels.

Method	R^2^	ΜAΕ	RMSE	Rank1
	15 min			
Proposed	0.98613	0.26120	0.4703	-
MLP ensemble	0.9852	0.2760	0.4869	-
MLP	0.98568	0.26936	0.4782	19.25%
RBF	0.98574	0.27095	0.4773	19.94%
LR	0.98562	0.26700	0.4793	10.49%
SVR	0.98541	0.26931	0.4829	14.23%
RF	0.98373	0.29531	0.5071	24.27%
SR	0.98561	0.26715	0.4795	11.82%
	1 h			
Proposed	0.93793	0.60224	0.9946	-
MLP ensemble	0.9344	0.6330	1.0240	-
MLP	0.91697	0.66794	1.1500	21.56%
RBF	0.93253	0.64235	1.0374	20.77%
LR	0.93168	0.64174	1.0438	8.91%
SVR	0.93008	0.65376	1.0562	10.70%
RF	0.92912	0.67311	1.0614	20.79%
SR	0.93045	0.64079	1.0532	17.27%
	2 h			
Proposed	0.88147	0.88279	1.3767	-
MLP ensemble	0.8838	0.8965	1.3721	-
MLP	0.84455	0.99479	1.5854	20.32%
RBF	0.87052	0.96255	1.4472	18.32%
LR	0.87233	0.93356	1.4377	11.20%
SVR	0.86949	0.93765	1.4537	11.38%
RF	0.86653	0.96596	1.4675	22.94%
SR	0.86953	0.93189	1.4534	15.92%
	3 h			
Proposed	0.84143	1.0599	1.5871	-
MLP ensemble	0.8359	1.0859	1.6192	-
MLP	0.78486	1.2504	1.8538	18.08%
RBF	0.82483	1.1367	1.6727	20.14%
LR	0.82241	1.1270	1.6843	8.74%
SVR	0.81914	1.1512	1.6997	11.82%
RF	0.81895	1.1391	1.7006	23.67%
SR	0.81893	1.1229	1.7007	17.54%
	6 h			
Proposed	0.83251	1.1144	1.6462	-
MLP ensemble	0.8272	1.1951	1.6888	-
MLP	0.83036	1.1758	1.6733	20.77%
RBF	0.80289	1.2848	1.8037	21.31%
LR	0.77800	1.3308	1.9141	10.34%
SVR	0.75400	1.4300	2.0150	16.42%
RF	0.81341	1.2119	1.7549	21.08%
SR	0.77373	1.3413	1.9325	10.08%
	24 h			
Proposed	0.78474	1.1835	1.8174	-
MLP ensemble	0.7827	1.2372	1.8468	-
MLP	0.78073	1.2313	1.8553	21.93%
RBF	0.73576	1.4119	2.0367	21.83%
LR	0.75712	1.3188	1.9526	11.16%
SVR	0.73669	1.3031	2.0331	16.82%
RF	0.76487	1.2694	1.9212	16.71%
SR	0.74761	1.3419	1.9905	11.56%

Note: The quality metrics of the proposed methodology are denoted by text in colour.

## Data Availability

The data are not publicly available due to confidentiality and privacy reasons.

## Nomenclature

AP	active power
LR	linear regression
ML	Machine-learning
MAE	mean absolute error
MLP	multi-layer perceptron
NN	neural network
RBF	radial basis function
RF	random forests
RES	renewable energy sources
SBL	sparse Bayesian learning
SR	sparse regression
SVR	support vector regression
